# microRNA let‐7g suppresses PDGF‐induced conversion of vascular smooth muscle cell into the synthetic phenotype

**DOI:** 10.1111/jcmm.13269

**Published:** 2017-07-12

**Authors:** Tzu‐Ming Wang, Ku‐Chung Chen, Po‐Yuan Hsu, Hsiu‐Fen Lin, Yung‐Song Wang, Chien‐Yuan Chen, Yi‐Chu Liao, Suh‐Hang H. Juo

**Affiliations:** ^1^ Department of Medical Research China Medical University Hospital Taichung Taiwan; ^2^ Department of Biochemistry and Molecular Cell Biology School of Medicine College of Medicine Taipei Medical University Taipei Taiwan; ^3^ Department of Neurology Kaohsiung Medical University Kaohsiung Taiwan; ^4^ Department of Life Science National Taiwan University Taipei Taiwan; ^5^ Institute of Fisheries Science National Taiwan University Taipei Taiwan; ^6^ Graduate Institute of Medicine Kaohsiung Medical University Kaohsiung Taiwan; ^7^ Department of Neurology National Yang‐Ming University School of Medicine Taipei Taiwan; ^8^ Department of Neurology Taipei Veterans General Hospital Taipei Taiwan; ^9^ Graduate Institute of Biomedical Sciences China Medical University Taichung Taiwan; ^10^ Institute of New Drug Development China Medical University Taichung Taiwan; ^11^ Brain disease research center China Medical University Taichung Taiwan

**Keywords:** microRNA let‐7g, PDGF, vascular smooth muscle cell, α‐SMA, calponin atherosclerosis

## Abstract

Platelet‐derived growth factor (PDGF) can promote vascular smooth muscle cells (VSMCs) to switch from the quiescent contractile phenotype to synthetic phenotype, which contributes to atherosclerosis. We aimed to investigate the role of microRNA let‐7g in phenotypic switching. Bioinformatics prediction was used to find let‐7g target genes in the PDGF/mitogen‐activated protein kinase kinase kinase 1 (MEKK1)/extracellular signal‐regulated kinase (ERK)/Krüppel‐like factor‐4 (KLF4) signalling pathway that affects VSMC phenotypic switching. The luciferase reporter assay and let‐7g transfection were used to confirm let‐7g target genes. Two contractile proteins alpha‐smooth muscle actin (α‐SMA) and calponin were VSMC‐specific genes and were measured as the indicators for VSMC phenotype. Lentivirus carrying the let‐7g gene was injected to apolipoprotein E knockout (apoE^−/−^) mice to confirm let‐7g's effect on preventing atherosclerosis. Through the PDGF/MEKK1/ERK/KLF4 signalling pathway, PDGF‐BB can inhibit α‐SMA and calponin. The PDGFB and MEKK1 genes were predicted to harbour let‐7g binding sites, which were confirmed by our reporter assays. Transfection of let‐7g to VSMC also reduced PDGFB and MEKK1 levels. Moreover, we showed that let‐7g decreased phosphorylated‐ERK1/2 while had no effect on total ERK1/2. KLF4 can reduce VSMC‐specific gene expression by preventing myocardin–serum response factor (SRF) complex from associating with these gene promoters. The immunoprecipitation assay showed that let‐7g decreased the interaction between KLF4 and SRF. Further experiments demonstrated that let‐7g can increase α‐SMA and calponin levels to maintain VSMC in the contractile status. Injection of lentivirus carrying let‐7g gene increased let‐7g's levels in aorta and significantly decreased atherosclerotic plaques in the apoE^−/−^ mice. We demonstrated that let‐7g reduces the PDGF/MEKK1/ERK/KLF4 signalling to maintain VSMC in the contractile status, which further reduce VSMC atherosclerotic change.

## Introduction

Proliferation and phenotypic switching of vascular smooth muscle cells (VSMCs) is an important pathogenesis for the development of atherosclerotic plaque 1. Oxidized low‐density lipoprotein (oxLDL) can induce platelet‐derived growth factor (PDGF) secretion 2 and increase PDGF receptor β (PDGFRβ) activation in VSMCs 3. Platelet‐derived growth factor exists as PDGF‐AA or PDGF‐BB homodimer, or PDGF‐AB heterodimer. It has also been found that PDGF‐BB and PDGFRβ are expressed in VSMCs within atherosclerotic lesions, and that inhibition of PDGF‐BB and PDGFRβ reduces atherosclerotic lesion size 4, 5, 6, 7, 8, 9. Platelet‐derived growth factor can promote VSMCs to switch from the quiescent contractile phenotype (also known as differentiated state) to the ‘synthetic’ phenotype (also known as dedifferentiated state) by inhibiting expression of VSMC‐specific marker genes such as alpha‐smooth muscle actin (α‐SMA) and calponin 10, 11. Vascular smooth muscle cells of synthetic phenotype is a key element in atherosclerotic development because this type of VSMCs can migrate, proliferate and generate extracellular matrix proteins 12.

The switching of VSMC phenotypes between the contractile type and synthetic type is regulated by Krüppel‐like factor‐4 (KLF4), myocardin and serum response factor (SRF). Myocardin is a coactivator of SRF and KLF4 can affect both myocardin and SRF. Krüppel‐like factor‐4 can suppress myocardin expression 13. In addition, KLF4 can also inhibit myocardin/SRF complex from binding to the CArG box 13 that is found in the promoters of most VSMC‐specific marker genes. For example, the expression of α‐SMA and calponin that are VSMC‐specific contractile proteins is regulated by myocardin and SRF 14. Platelet‐derived growth factor‐BB up‐regulates KLF4 through the mitogen‐activated protein kinase kinase kinase 1 (MEKK1)/extracellular signal‐regulated kinase (ERK)/SP‐1 pathway 15, 16. Accordingly, PDGF‐BB mediates VSMC phenotypic switching through down‐regulation of myocardin 15.

MicroRNAs (miRNAs) are a family of small (~22 nucleotides) noncoding RNA that negatively regulate the expression of target genes 17. Previous studies from our group have shown that microRNA let‐7g can prevent the uptake of oxLDL into VSMC by inhibiting LOX‐1 expression 18. Our group also demonstrated that let‐7g can improve endothelial functions by targeting the transforming growth factor β (TGF‐β) pathway and by increasing sirtuin‐1 (SIRT‐1) expression 19. This study aimed to examine let‐7g's roles in the PDGF/MEKK1/ERK/KLF4 pathway and in regulating VSMC‐specific marker genes during VSMC phenotypic switching.

## Materials and methods

### Materials

Primary human aortic smooth muscle cells (HASMC), medium 231 and smooth muscle cell growth supplement (SMGS) were purchased from Cascade Biologics (Portland, OR, USA). Other cell culture‐related reagents were purchased from GIBCO‐BRL (Grand Island, NY, USA). Trizol^®^ reagent, Lipofectamine 2000, secondary antibodies were purchased from Invitrogen (Carlsbad, CA, USA). SYBR^®^ Green PCR Master Mix, MultiScribe(tm) Reverse Transcriptase Kit, TaqMan^®^ let‐7g and U44 Assays and let‐7g mimic were purchased from Applied Biosystems (Foster City, CA, USA). Primer sets were synthesized from Mission Biotech. Protein G plus/protein A‐agarose and PCR Master Mix were purchased from Thermo Fisher Scientific Inc (Waltham, MA, USA). Unless otherwise specified, all other reagents were of analytical grade. Recombinant human PDGF‐BB was purchased from R&D Systems (Minneapolis, MN, USA). Antibodies against α‐tubulin, α‐SMA and calponin were purchased from Abcam (Cambridge, UK). Antibodies recognizing phospho‐ERK1/2 (Thr202/Tyr204) and ERK1/2 were purchased from Santa Cruz Biotechnology (Santa Cruz, CA, USA). Antibodies against KLF4 and SRF were obtained from Proteintech (Chicago, IL, USA).

### Let‐7g expressing lentivirus

A lentivirus vector named pCDH‐CMV‐MCS‐EF1 containing a CMV‐driven EGFP reporter was purchased from LabLife Co., Ltd. (Cambridge, MA, USA). The full‐length sequence of let‐7g was amplified from human genomic DNA by PCR. Primer pairs for constructing lentivirus vectors were let‐7g forward: TATGAATTCAGCTTTGCTGCCAAGCCTCTGCTGTG and let‐7g reverse: TATGGATCCCCTAAGAAGAAAAAGACTTCCTCCCC. The PCR products were annealed and inserted between the EcoRI and BamHI sites of the plasmid. The positive clones were identified as lentiviral vectors and pCDH‐let‐7g. Correct insertions of pCDH‐let‐7g plasmids were confirmed by restriction mapping and DNA sequencing to ensure accuracy.

### Lentivirus production

Recombinant lentiviruses were produced by cotransfecting 293T cells with the lentivirus expression plasmid and packaging plasmids using Lipofectamine 2000. Infectious lentiviruses were harvested at 72 hrs post‐transfection, centrifuged to eliminate cell debris and then filtered through 0.22‐μm cellulose acetate filters (Millipore, Billerica, MA, USA). Infectious titre was determined by counting GFP positive‐293T cells using the fluorescence microscope. Virus titres were at the range of 10^8^ transducing units/ml medium. Determination of the lentiviral titre allowed us to estimate the multiplicity of infection (MOI) and thus to deduce the infectious activity of the viral stocks. The pCDH mock vector was also packaged and used as a negative control, which has no significant homology to mouse gene sequences.

### Lentiviral vector transduction in cultured HASMCs

On the day of transduction, cells were replated at 5 × 10^3^ cells/well in 96‐well plates along with recombinant lentivirus encoding for let‐7g gene at different MOIs in serum‐free growth medium containing 5 mg/ml polybrene at 37°C and 5% CO_2_. After 4 hrs, serum containing growth medium was added to the cells, and there was complete replacement of growth medium after 48 hrs. Then, after days post‐transfection, reporter gene expression was examined using fluorescent microscopy and quantitative real‐time PCR (qPCR).

### Apolipoprotein E knockout mice study

The apolipoprotein E knockout (apoE^−/−^) mice were originally purchased from Jackson Laboratory (Bar Harbor, MI, USA). ApoE^−/−^ mice aged 8 weeks were fed with the Western diet containing 0.25% cholesterol and 15% cacao butter (Testdiet^®^ 5TJN), and were allocated to receive either the pCDH‐let‐7g (*n* = 6) treatment or placebo PBS/control lentivirus (*n* = 6) by tail vein hydrodynamic injection (1 × 10^7^ TU diluted in 1% w/v body weight PBS, typically 0.2–0.25 ml). Mice were injected once per week for 12 weeks. Weight gain was monitored every week, and food intake was monitored twice daily during the period of each study. Mice were maintained in a temperature‐controlled (25°C) facility with a strict 12‐hr light: dark cycle. All animals were provided free access to food and water throughout the experiment.

After the 12‐week treatment, mice were killed using an overdose of isoflurane anaesthesia. Mice were perfused with PBS, followed by 10% sucrose in PBS. The blood and aorta were collected for further studies. The blood biochemical tests were conducted by Kaohsiung Medical University Hospital. To detect atherosclerotic plaques, aortas were stained by oil red, and the lesion areas were quantified by densitometer. For measuring the let‐7g levels, total RNA was extracted from aorta tissues. Let‐7g and U6B levels were, respectively, determined by quantitative real‐time PCR. The U6B levels were used as internal control.

### Cell culture and transfection

Human aortic smooth muscle cells were grown in medium 231 supplemented with SMGS at 37°C in a humidified atmosphere of 95% air/5% CO_2_. Cells between passages 6 and 10 were used in all experiments. For transfection experiment, cells were seeded onto six‐well plate at a density of 2 × 10^5^ cells/well. Cells achieving 95% confluence were transfected with let‐7g mimic or let‐7g inhibitor using the HiPerFect Transfection Reagent (Qiagen, Hilden, Germany).

### RNA isolation and qPCR

Total RNA extraction was carried out using Trizol^®^ according to the manufacturer instructions. RNA quality was confirmed using A260/A280 readings. The cDNA was synthesized from 1 μg total RNA using a random primer and the MultiScribe(tm) Reverse Transcriptase Kit. For quantitative real‐time PCR, specific primers for human or mouse MEKK1, PDGFB, α‐SMA, calponin and GAPDH are listed in Table S1. For let‐7g and U44 detection, cDNA was synthesized from TaqMan^®^ MicroRNA Assays. The cDNA was diluted 1:30 with PCR grade water and stored at −20°C. Relative quantification of gene expression was performed with pre‐optimized conditions using the ABI 7500 real‐time PCR machine (Applied Biosystems). The expression ratios were calculated as the normalized CT difference between the control and pCDH‐let‐7g with the adjustment for the amplification efficiency relative to the expression level of the U44.

### Luciferase reporter assay

To conduct the luciferase reporter assay, we amplified the PDGFB and MEKK1 3′‐untranslated region (3′‐UTR) regions by PCR using specific primers of which the forward primer was SpeI‐site‐linked and the reverse primer MluI‐site‐linked. Human aortic smooth muscle cell genomic DNA was used as the template. PCR products were digested with SpeI and MluI and cloned downstream of the luciferase gene in the pMIR‐REPORT luciferase vector (Ambion, Austin, TX, USA). These vectors were sequenced and named pMIR‐PDGFB‐3UTR and pMIR‐MEKK1‐3UTR. To further make sure that let‐7g directly targets to the PDGFB and MEKK1 3′‐UTR, we conducted the site‐directed mutagenesis to mutate the let‐7g target site in the PDGFB and MEKK1 3′‐UTR. The site‐directed mutagenesis was carried out using the QuikChangeH Site‐Directed Mutagenesis Kit (Stratagene, La Jolla, CA, USA). The pMIR‐PDGFB‐3UTR and pMIR‐MEKK1‐3UTR were used as a template. For reporter assays, the cells were transiently transfected with wild‐type or mutant reporter plasmid and let‐7g expressing lentivirus using Lipofectamine 2000. pEGFP plasmids were cotransfected and acted as the internal control. Reporter assay was performed at 24 hrs post‐transfection using the Luciferase Assay System (Promega, Madison, WI, USA).

### Western blot analysis

For the Western blot analysis, HASMCs were lysed with buffer containing 20 mM Tris–HCl (pH 7.4), 150 mM NaCl, 1% Tween 20, 0.1% SDS, 1 mM ethylenediaminetetraacetic acid and complete protease inhibitor cocktail (Roche Applied Science, Mannheim, Germany). Twenty micrograms of proteins was subject to SDS‐PAGE, and then, electrophoresed proteins were transferred to polyvinylidene difluoridemembrane (Millipore). The immunoblots were incubated with primary antibodies overnight at 4°C. After washing, the immunoblots were incubated with horseradish peroxidase‐conjugated secondary antibodies for 1 hr at 4°C. The peroxidase‐conjugated secondary antibody was visualized using enhanced chemiluminescence (ECL) chemiluminescent detection system (PerkinElmer, Shelton, CT, USA) according to the manufacturer's instructions.

### Immunofluorescence microscopy

Human aortic smooth muscle cells were treated with PDGF‐BB (5 ng/ml) for 48 hrs before let‐7g transfection (5 nM) for 24 hrs on poly (l‐lysine)‐coated glass coverslips in 6‐well plates. Then, cells were fixed in 4% formaldehyde for 15 min. and permeabilized with 0.2% Triton X‐100 for 5 min. The coverslips were washed with 0.05% TBST three times and blocked with 5% bovine serum albumin for 1 hr at 37°C. Immunofluorescence staining was performed using anti‐calponin (1:200) and anti‐α‐SMA (1:200) antibodies. The cells were counterstained with 4′,6‐diamidino‐2‐phenylindole (DAPI) to identify the nuclei.

### Co‐immunoprecipitation assay

Human aortic smooth muscle cell extract was precipitated with 1 μg of KLF4 or 1 μg of SRF antibodies for 4 hrs at 4°C, and antibody–protein complex was incubated with protein G plus/protein A‐agarose (Santa Cruz Biotechnology) for overnight at 4°C. The immune complexes were centrifuged and washed four times with buffer containing 20 mM Tris‐HCl, 150 mM NaCl, 0.1% Triton X‐100 and 10% glycerol. Agarose bead was then subject to the Western blotting analyses.

### Statistical analysis

Student's *t*‐test was used to compare all experimental results. A *P*‐value less than 0.05 was considered significant.

## Result

### Let‐7g down‐regulates PDGF‐BB‐activated MEKK1/ERK/KLF4 pathway

**Figure 1:**
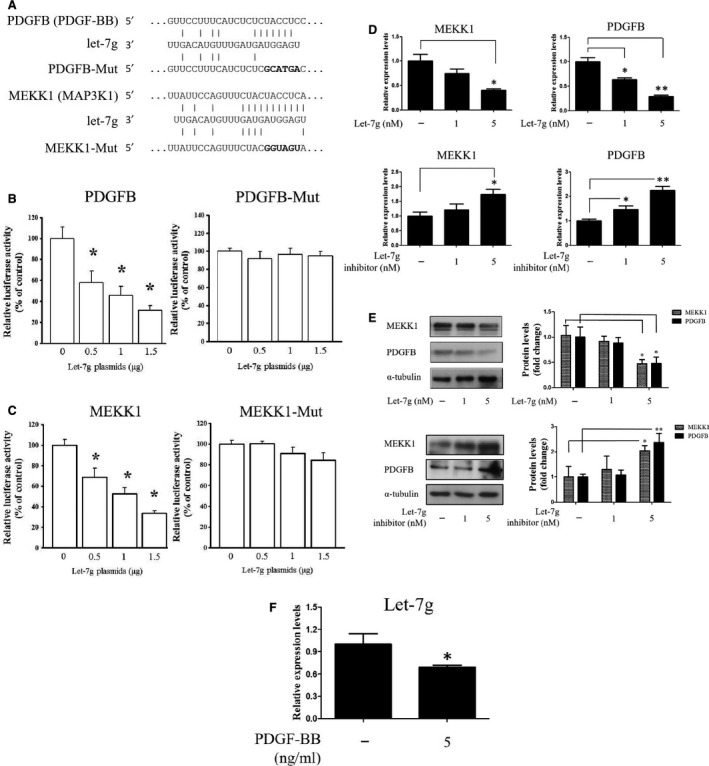
Identification of PDGFB and MEKK1 as let‐7g direct target genes. (**A**) Schematic diagram shows the let‐7g‐binding site in PDGFB and MEKK1 3′‐UTR regions. (**B** and **C**) The effects of let‐7g on luciferase activity. Cells were transfected with pMIR‐REPORT luciferase vector that carries wild‐type or mutant type PDGFB and MEKK1 3′‐UTR, and let‐7g‐expressing vector. The luciferase activity was measured at 24 hrs in triplicate. (**D** and **E**) Cells were transfected with let‐7g or its inhibitor, and gene expression was measured at 24 hrs. The mRNA and protein levels of MEKK1 and PDGFB were reduced by let‐7g and increased by let‐7g inhibitor. (**F**) The let‐7g level was determined in the PDGF‐BB‐treated cells. Data are means ± S.E.M. from three experiments; *P < 0.05; **P < 0.01.

Through the PDGF/MEKK1/ERK/KLF4 signalling pathway, PDGF‐BB can inhibit myocardin‐SRF complex from binding bind to the CArG box in the promoters of VSMC‐specific genes 20, 21. Based on bioinformatics prediction, potential let‐7g binding sites may exist in the 3′‐UTRs of the PDGFB and MEKK1 genes (Fig. 1A). Using the reporter assays, we showed that let‐7g could directly target wild‐type sequences of PDGFB and MEKK1 genes and suppress the luciferase activity, while let‐7g had no effects on the mutant sequences (Fig. 1B and C).

To further confirm that let‐7g's knockdown effect on these 2 genes, transient transfection of let‐7g mimic into HASMCs caused a reduction of their protein and mRNA levels at 24 hrs, whereas transfection of let‐7g inhibitor caused the opposite effect (Fig. 1D and E). These results indicated that MEKK1 and PDGFB are direct targets of let‐7g in human cells. It has been shown that let‐7g expression was altered in PDGF‐treated glioblastoma cells 22. In our model, let‐7g expression was suppressed in PDGF‐BB‐treated HASMCs (Fig. 1F).

**Figure 2:**
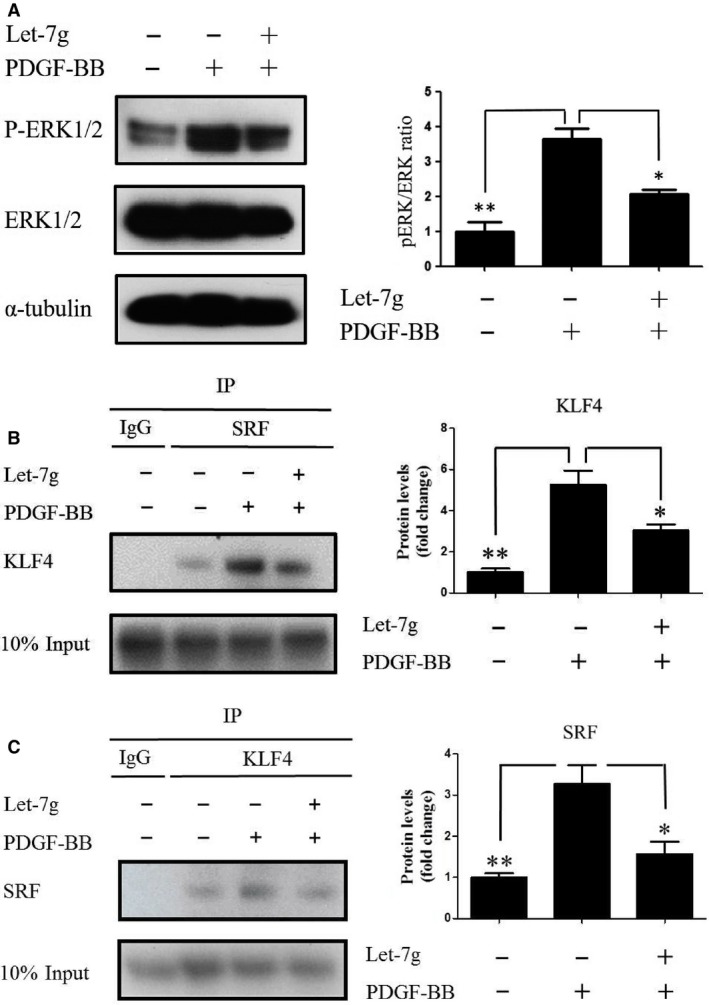
Let‐7g inhibits PDGF‐induced activation of ERK1/2 and KLF4. HASMCs were incubated with PDGF‐BB (5 ng/ml) for 48 hrs before the transfection of let‐7g (5 nM) for 24 hrs. The cell extracts were analysed. (**A**) Western blot was used to detect phosphorylated ERK1/2 and total ERK1/2. (**B** and **C**) Co‐immunoprecipitation assay was used to test for the interaction between KLF4 and SRF. Data represent mean ± S.E.M. of three independent experiments. *P < 0.05; **P < 0.01.

Because MEKK1 can regulate ERK1 and ERK2 (ERK1/2) 23, we then investigated the effect of let‐7g on the PBDGF‐BB‐induced phosphorylation of ERK1/2. As shown in Figure 2A, let‐7g reduced phosphorylated‐ERK1/2 while had no effect on total ERK1/2. Previous studies have reported that ERK1/2 are the upstream kinases of KLF4 21 and KLF4 can down‐regulate myocardin and prevent myocardin‐SRF complex from associating with VSMC‐specific gene promoters 16. We therefore conducted the co‐immunoprecipitation assay to test whether let‐7g inhibits the interaction between SRF and KLF4. The result showed that let‐7g decreased the interaction between KLF4 and SRF in the presence of PDGF‐BB (Fig. 2B and C).

### Effects of let‐7g on α‐SMA and calponin expression in PDGF‐BB‐treated HASMCs

**Figure 3:**
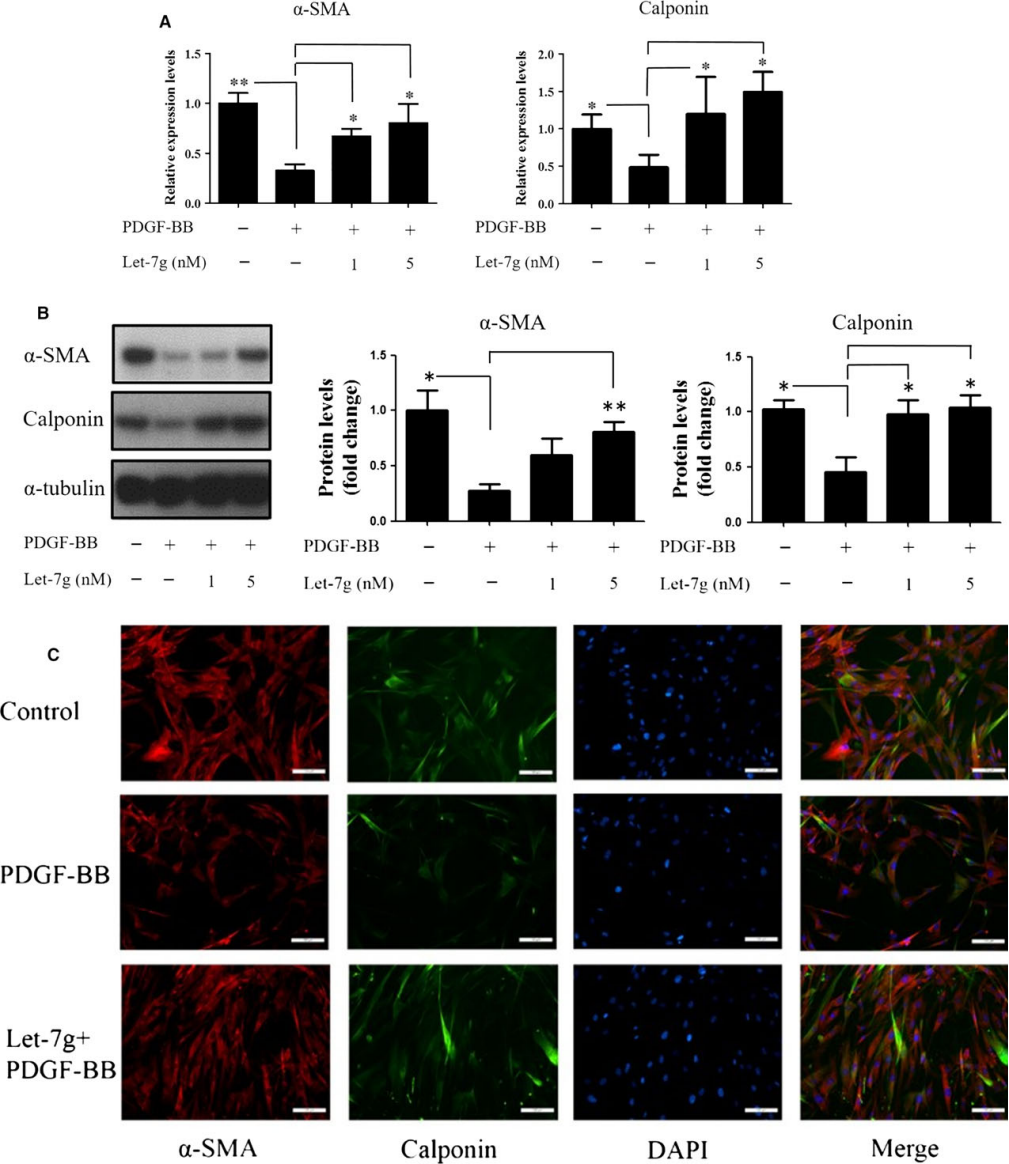
Let‐7g effect on α‐SMA and calponin expression in PDGF‐BB‐treated HASMCs. Cells were incubated with PDGF‐BB (5 ng/ml) for 48 hrs and then transfected with let‐7g (1 or 5 nM) for another 24 hrs. (**A**) The mRNA levels of α‐SMA and calponin were measured by real‐time PCR. (**B**) The protein levels of α‐SMA and calponin were determined by Western blot. Data are presented as mean ± S.E.M. from three independent experiments, and each experiment was performed in triplicate. *P < 0.05 and **P < 0.01 *versus* the negative control group. The entire Western blotting analysis of α‐SMA and calponin is shown in Figure S2 (**C**) Immunofluorescent stain was used to detect α‐SMA and calponin in HASMCs treated with PDGF‐BB along with let‐7g. Nuclei were stained with DAPI (blue). Scale bars, 40 μm.

We further tested whether let‐7g can maintain VSMC in the contractile status by measuring α‐SMA and calponin levels in the cells. Our data showed that PDGF‐BB decreased α‐SMA and calponin mRNA levels, whereas let‐7g could dose dependently restore their expression (Fig. 3A). The protein data and immunofluorescence imaging analysis also showed the consistent results (Fig. 3B and 3C, Fig. S2). Taken together, these findings indicate that let‐7g can restore PDGF‐BB‐reduced VSMC marker gene expression, and therefore, let‐7g can prevent VSMC from atherosclerotic change.

### Let‐7g reduces atherosclerotic plaque formation

**Figure 4:**
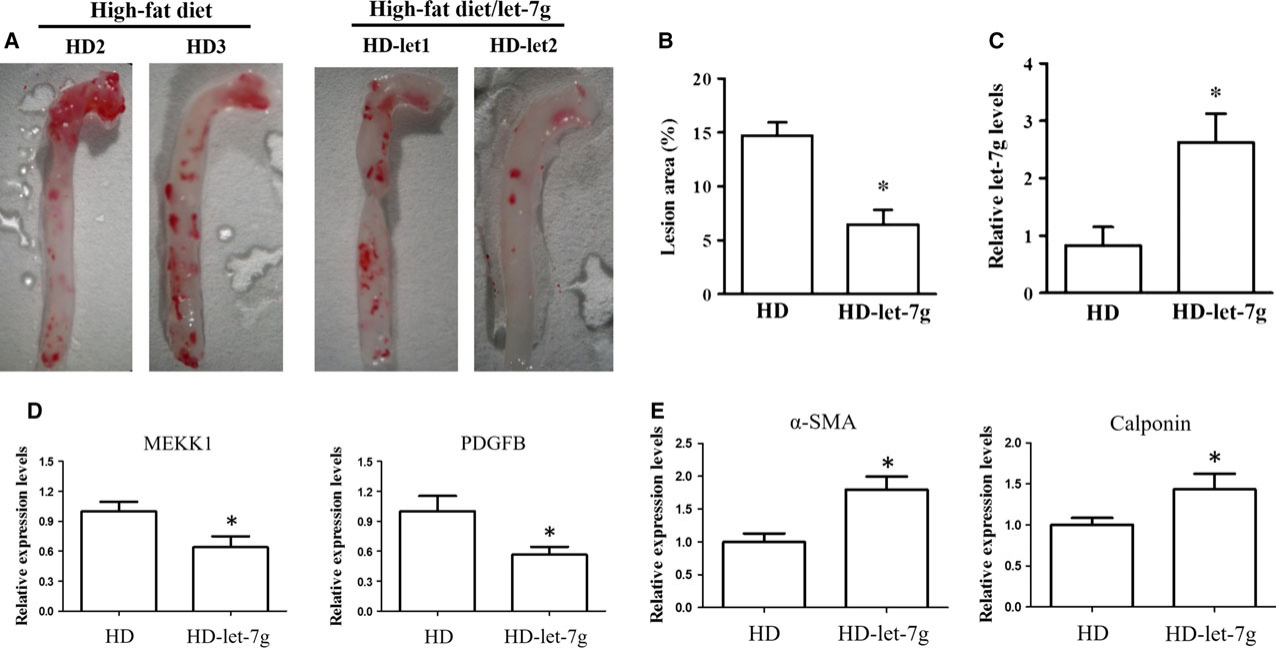
Effect of let‐7g on atherosclerotic plaque formation. (**A**) The atherosclerotic plaques in the aortas of apoE^−/−^ mice under a high‐fat diet. (**B**) The quantitative result of atherosclerotic areas and (**C**) let‐7g levels in the aortas. (**D** and **E**) The mRNA levels of MEKK1, PDGFB, α‐SMA and calponin in aortas were determined by qPCR. *N* = 6 for each group, * means *P* < 0.05. The data presented as mean ± S.E.

To test for the effect of let‐7g on prevention of atherosclerosis *in vivo*, apoE^−/−^ mice under a high‐fat diet were injected lentivirus carrying either the let‐7g‐expressing vector or negative control vector for 12 weeks. The atherosclerotic areas in the entire aorta were decreased in mice treated with let‐7g compared to those in the mice treated with placebo (Fig. 4A and B). Let‐7g treatment indeed increased let‐7g levels by 3.6‐fold in the aortas (Fig. 4C). These results confirm that let‐7g suppressed atherosclerotic plaque formation. The mRNAs levels of PDGFB, MEKK1, α‐SMA and calponin from entire aorta were measured. Our *in vivo* results showed that PDGFB and MEKK1 mRNA levels were suppressed by let‐7g treatment (Fig. 4D), while α‐SMA and calponin mRNA levels were increased by let‐7g treatment (Fig. 4E).

### Let‐7g decreases body weight and improves lipid profile

The let‐7g‐treated apoE^−/−^ mice had slower gain of body weight than the placebo‐treated mice when all mice were under a high‐fat diet (Fig. S1A). The placebo‐treated mice had twofold gain of body weight than the let‐7g‐treated mice in the end of 12‐week experiment (Fig. S1B). Fasting blood sugar, the lipid profile and liver function glutamic‐pyruvic transaminase (also known as alanine aminotransferase, ALT) levels were significantly better in the let‐7g‐treated apoE^−/−^ mice than in the placebo‐treated apoE^−/−^ mice in the end of the 12‐week experiment (Fig. [Link], [Link]).

## Discussion

**Figure 5:**
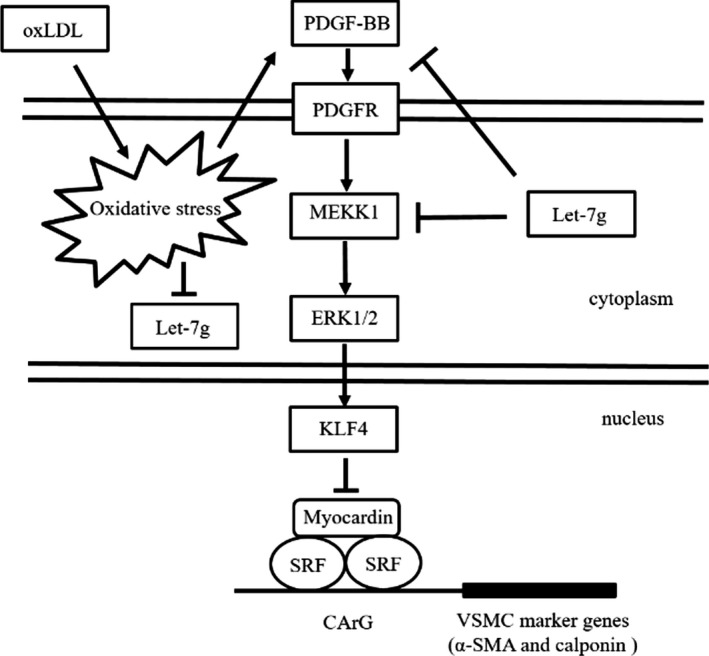
Schematic diagram shows that let‐7g affects PDGF/MEKK1/ERK/KLF4 signalling.

The present study demonstrated that let‐7g has multiple inhibitory effects on the PDGF signalling in VSMCs. First, let‐7g could directly down‐regulate the PDGFB and MEKK1 genes leading to reduced interaction between KLF4 and SRF. Secondly, both *in vitro* and *in vivo* data showed that let‐7g could keep VSMCs in the contractile status by restoring α‐SMA and calponin expression. The major findings in the present study are schematically summarized in Figure 5. In addition, the present study also showed that let‐7g could reduce the formation of atherosclerotic lesions in apoE^−/−^ mice under a high‐fat diet. Apart from our previous findings regarding let‐7g's effect on LOX‐1 and TGF‐β signalling 18, 19, here we further revealed another mechanism accounting for let‐7g beneficial effects.

It is known that PDGF‐BB can cause VSMC phenotypic switching from the contractile type to the synthetic type 24. Disruption of PDGF‐BB signalling has been shown to delay or prevent atherogenesis 8. Our data showed that α‐SMA and calponin were down‐regulated in PDGF‐BB‐treated HASMCs, but overexpression of let‐7g could restore their expression. MEKK1 is involved in the PDGF‐BB‐induced VSMC phenotypic switching 25, and we confirmed PDGFB and MEKK1 were let‐7g target genes. A recent study also demonstrated the direct effect of let‐7g on MEKK1 26. The myocardin/SRF complex is a key component to maintain the VSMC contractile status. Activated form of KLF4 can interact with SRF and also repress myocardin 13. Previous studies demonstrated that PDGF‐BB suppresses the promoter activity of VSMC marker genes *via* regulation of KLF4 21. ERK1/2 are the upstream kinases of KLF4 in the PDGF‐BB‐induced signalling pathway 21, 27. Here, we showed that let‐7g reduced the levels of phosphorylated‐ERK1/2 in PDGF‐BB‐treated HASMCs. Through the direct knockdown of PDGFB and MEKK1, let‐7g eventually suppressed the interaction between KLF4 and myocardin/SRF. These results supported let‐7g as an important regulator to maintain VSMC in the contractile status.

A recent study of PDGF/PDGFRβ signalling in VSMCs revealed that activation of this pathway increased inflammation but not atherosclerosis under a normal cholesterol level 6. However, the same authors further demonstrated that the PDGF signalling amplified hypercholesterolaemia‐induced atherosclerosis 6. The current study showed that let‐7g could simultaneously reduce cholesterol levels and block PDGF/PDGFRβ signalling in VSMCs. Furthermore, we have found that let‐7g decreases PDGF‐BB‐induced inflammatory genes (TNF‐α, IL‐8, IL‐1β and granulocyte‐macrophage colony‐stimulating factor (GM‐CSF)) expression in HASMCs (Fig. S3). Our previous studies also demonstrated that let‐7g could reduce inflammation by suppressing TGF‐β signalling in endothelial cells and knock down LOX‐1, both of which further augment the importance of let‐7g's role in preventing atherosclerosis. Negative feedback regulation plays an important role in homoeostasis. Our results showed that let‐7g can directly suppress PDGFB and inhibit PDGF‐BB‐activated MEKK1/ERK/KLF4 pathway in VSMCs, while PDGF‐BB can reduce let‐7g expression. Accordingly, a negative feedback regulation exists between let‐7g and PDGF‐BB. The possible mechanism of PDGF‐BB reduces let‐7g expression was showed in Figure S4

The present study found that let‐7g could improve the lipid profile in mice under high‐fat diet. Our group previously reported that let‐7g can up‐regulate SIRT‐1 expression 19, and SIRT‐1 has been indicated to play an important role in lipid and glucose metabolism 28, 29, 30, 31. Fasting can increase SIRT‐1 that further interacts with and deacetylates PGC‐1α in liver leading to improve glucose homoeostasis 28. The liver X receptor (LXR) mediates HDL synthesis, and SIRT‐1 plays an important role in cholesterol homoeostasis by regulating LXR 29. Specific knockout of SIRT‐1 in the liver (SIRT1 LKO) decreases fatty acid oxidation, which leads to body weight gain and the development of hepatic steatosis 30. In addition, the serum levels of cholesterol and LDL were increased in SIRT1 LKO mice on a lithogenic diet 31. Accordingly, let‐7g effect on SIRT‐1 may partially explain why let‐7g treatment can improve lipid profile and sugar level.

There are some limitations in the present study. We did not examine the atherosclerotic changes in the carotid artery. However, previous studies have shown that atherosclerotic changes are similar between carotid artery and aorta 32, 33 in the animal experiments. Therefore, we assumed that the results should be comparable between aortic lesion and carotid lesion in our apoE^−/−^ mice. As our present study focuses on VSMC changes, we did not conduct in‐depth studies to investigate the mechanisms for the change of biochemistry data and body weight in the animal study.

In summary, let‐7g inhibits formation of atherosclerotic lesions *via* regulation of multiple mechanisms. Our *in vivo* study showed that let‐7g improves lipid profile and sugar level in mice under a high‐fat diet. Our *in vitro* study demonstrated that let‐7g substantially inhibits the phenotypic switching of VSMCs, which is mediated through targeting PDGFB and MEKK1 genes. As contractile VSMCs are required to maintain normal functions arterioles and to prevent atherosclerosis, maintaining a sufficient level of let‐7g may reduce a risk for cardiovascular diseases.

## Conflicts of interest

The authors confirm that there are no conflicts of interest.

## Supporting information


**Table S1** PCR primer sequences.

**Figure S2** Entire Western blotting analysis of α‐SMA and calponin.

**Figure S3** Let‐7g inhibits inflammatory genes expression in PDGF‐BB‐treated HASMCs.

**Figure S4** Schematic diagram showing the possible mechanism of PDGF‐BB reduces let‐7g expression.
