# Supplementary material for: microRNA let‐7g suppresses PDGF‐induced conversion of vascular smooth muscle cell into the synthetic phenotype
Source: J Cell Mol Med. 2017 Jul 12;21(12):3592–601. doi: 10.1111/jcmm.13269 (PMC5706591; doi:10.1111/jcmm.13269)
Supplement: Supplementary file 1 — Table S1 PCR primer sequences. [file JCMM-21-3592-s013.docx]

Supplementary Table S. PCR primer sequences

| Primers | Sequence |
| --- | --- |
| Human MEKK1 F | 5′-TGCGGGCCAGACTGTACTTACT-3′ |
| Human MEKK1 R | 5′-TGCAGTTCTGAGGCCCAATAA-3′ |
| Human PDGFB F | 5′-ACTCGATCCGCTCCTTTGATGA-3′ |
| Human PDGFB R | 5′-GCTCGCCTCCAGAGTGGG-3′ |
| Human α-SMA F | 5′-AGCAGGCCAAGGGGCTATATAA-3′ |
| Human α-SMA R | 5′-CGTAGCTGTCTTTTTGTCCCATT-3′ |
| Human calponin F | 5′-GCAACTTCATCAAGGCCATCACCA-3′ |
| Human calponin R | 5′-TCGAATTTCCGCTCCTGCTTCTCT-3′ |
| Human TNFα F | 5′-TCTTCTCATTCCTGCTTGTGG-3' |
| Human TNFα R | 5′-GGTCTGGGCCATAGAACTGA-3′ |
| Human IL-1β F | 5′-CAGCTACGAATCTCCGACCAC-3′ |
| Human IL-1β R | 5′-GGCAGGGAACCAGCATCTTC-3′ |
| Human IL-8 F | 5′-ACTGAGAGTGATTGAGAGTGGAC-3′ |
| Human IL-8 R | 5′-AACCCTCTGCACCCAGTTTTC-3′ |
| Human GM-CSF F | 5′-TCTCAGAAATGTTTGACCTCCA-3′ |
| Human GM-CSF R | 5′-GCCCTTGAGCTTGGTGAG-3′ |
| Human GAPDH F | 5′-GTGAAGGTCGGAGTCAAC-3′ |
| Human GAPDH R | 5′-GTTGAGGTCAATGAAGGG-3′ |
| Mouse MEKK1 F | 5′-GTGGAGGACACTGTGGATGG-3′ |
| Mouse MEKK1 R | 5′-ATGGACTGTGTGCTCAAGGG-3′ |
| Mouse PDGFB F | 5′-ATGTGCCCTTCAGTCTGCTC-3′ |
| Mouse PDGFB R | 5′-GAGACAGGTCTCCTGCCCTA-3′ |
| Mouse α-SMA F | 5′-GGCATCCACGAAACCACCTA-3′ |
| Mouse α-SMA R | 5′-CTGTCAGCAATGCCTGGGTA-3′ |
| Mouse calponin F | 5′-GCAGGGCCCAACATAGAACT-3′ |
| Mouse calponin R | 5′-GGGTTTCTGGTTCTGGGAGG-3′ |
| Mouse GAPDH F | 5′-TGACCACAGTCCATGCCATC-3′ |
| Mouse GAPDH R | 5′-GACGGACACATTGGGGGTAG-3′ |
