# Supplementary figures and images for: microRNA let‐7g suppresses PDGF‐induced conversion of vascular smooth muscle cell into the synthetic phenotype
Source: J Cell Mol Med. 2017 Jul 12;21(12):3592–601. doi: 10.1111/jcmm.13269 (PMC5706591; doi:10.1111/jcmm.13269)

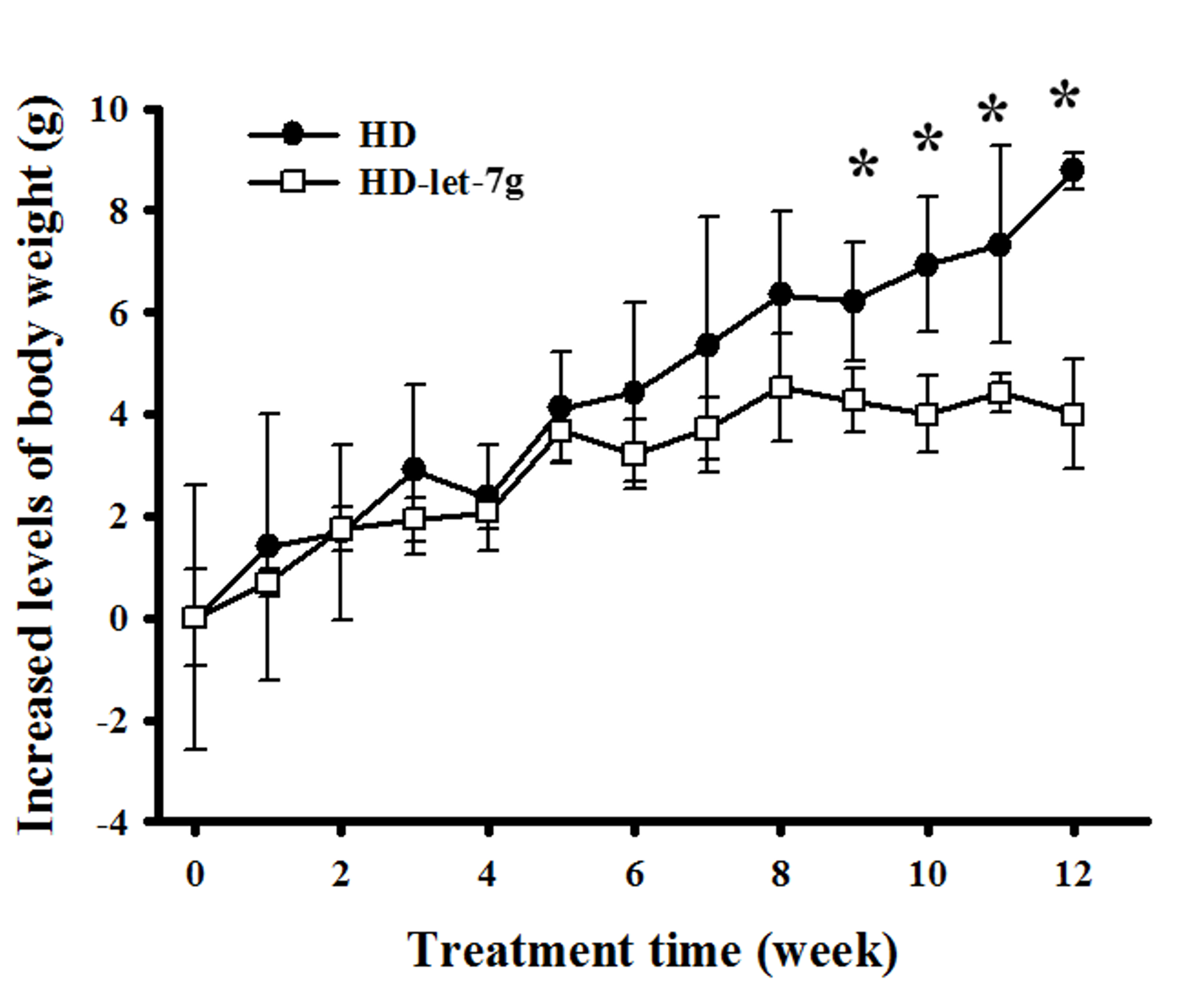

Supplement: Supplementary file 2 [file JCMM-21-3592-s001.tif]

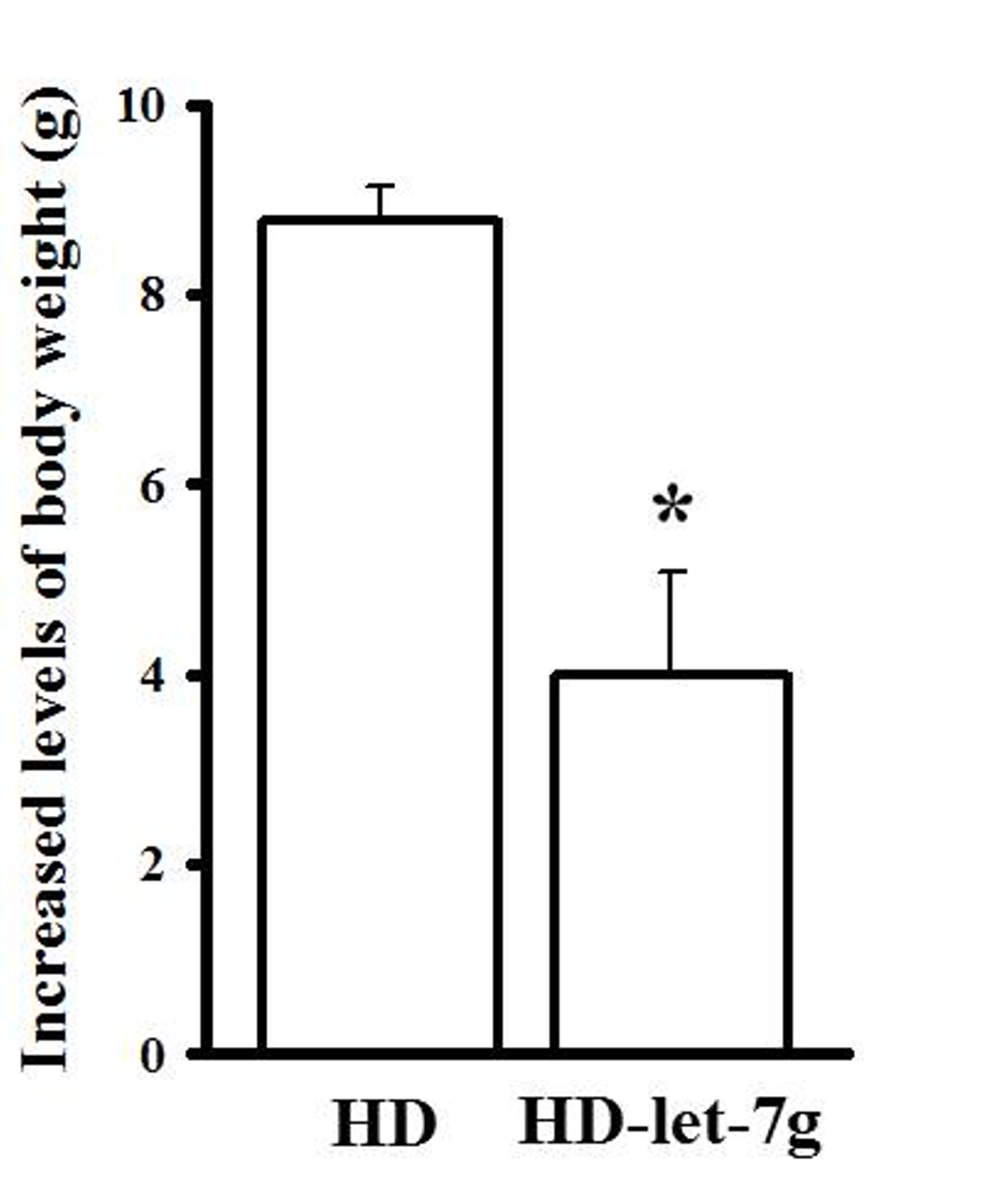

Supplement: Supplementary file 3 [file JCMM-21-3592-s002.tif]

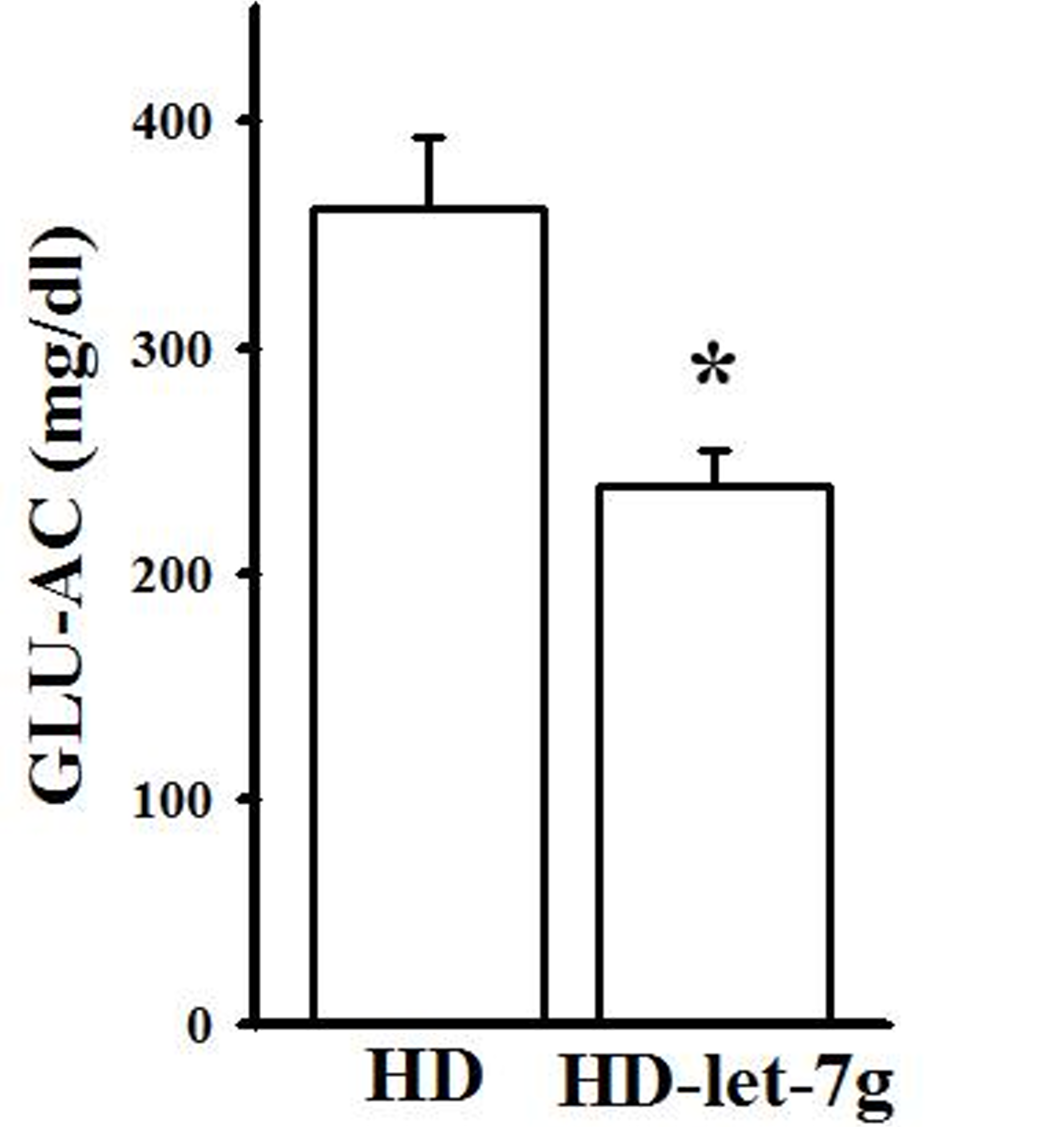

Supplement: Supplementary file 4 [file JCMM-21-3592-s003.tif]

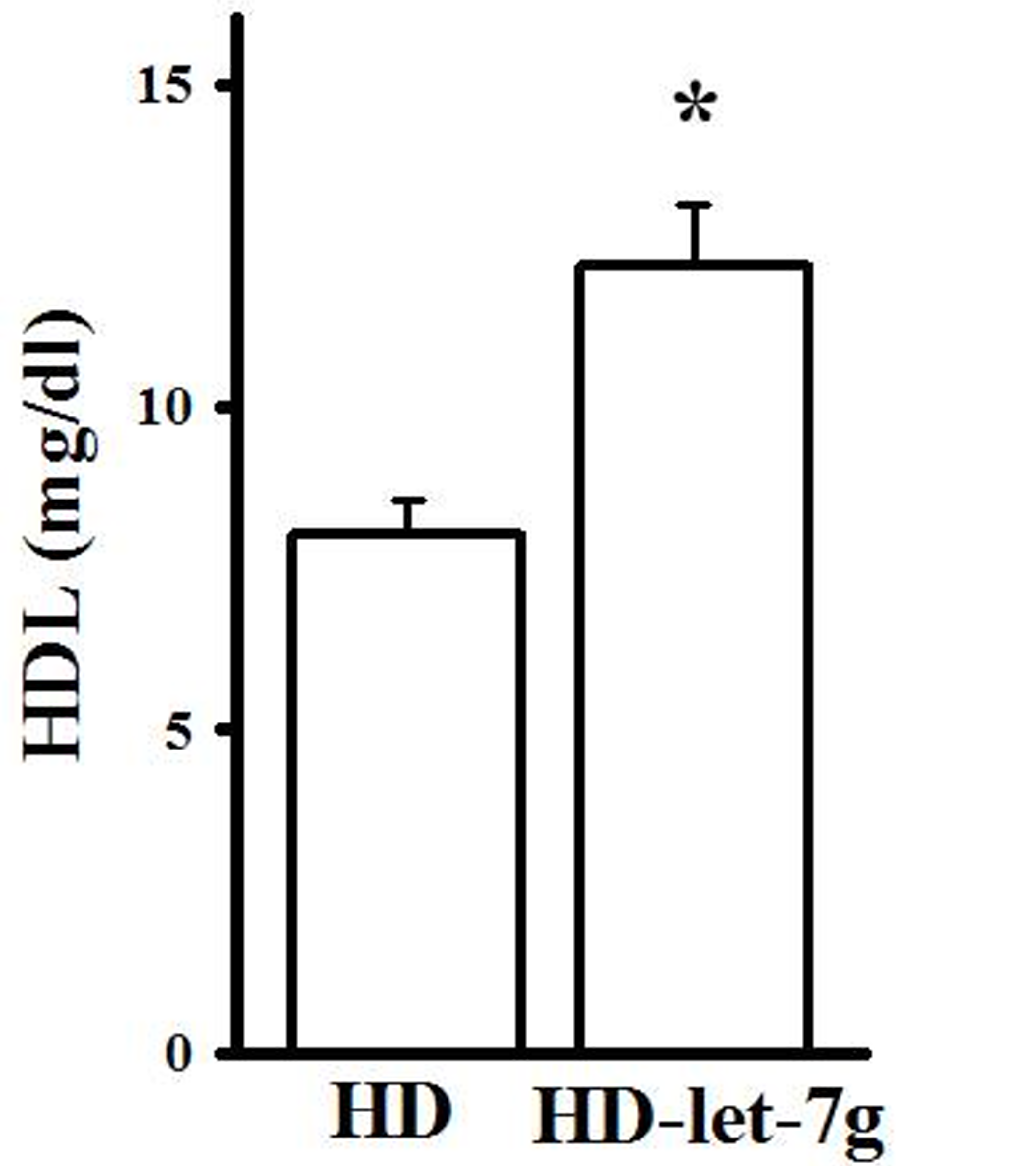

Supplement: Supplementary file 5 [file JCMM-21-3592-s004.tif]

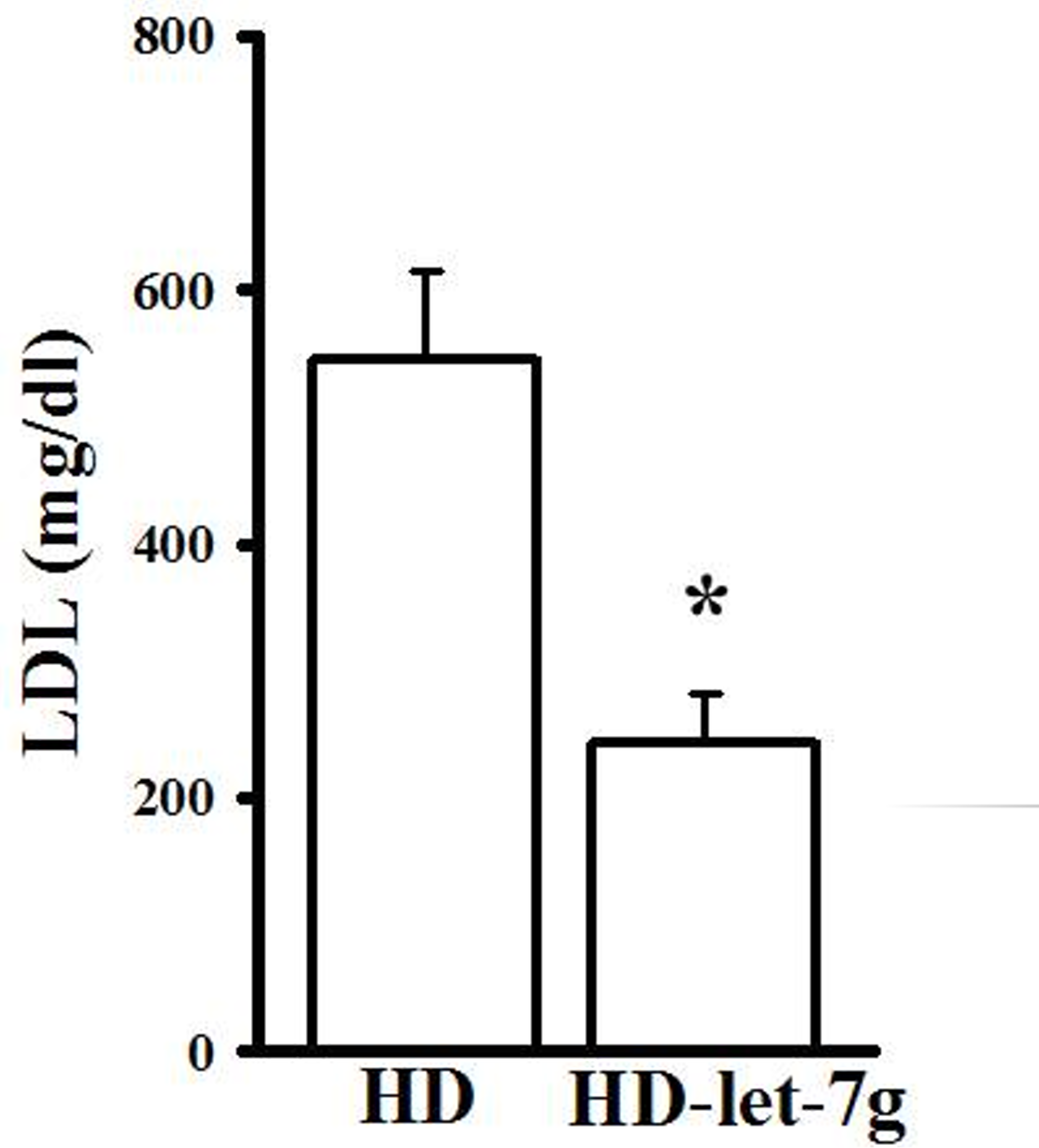

Supplement: Supplementary file 6 [file JCMM-21-3592-s005.tif]

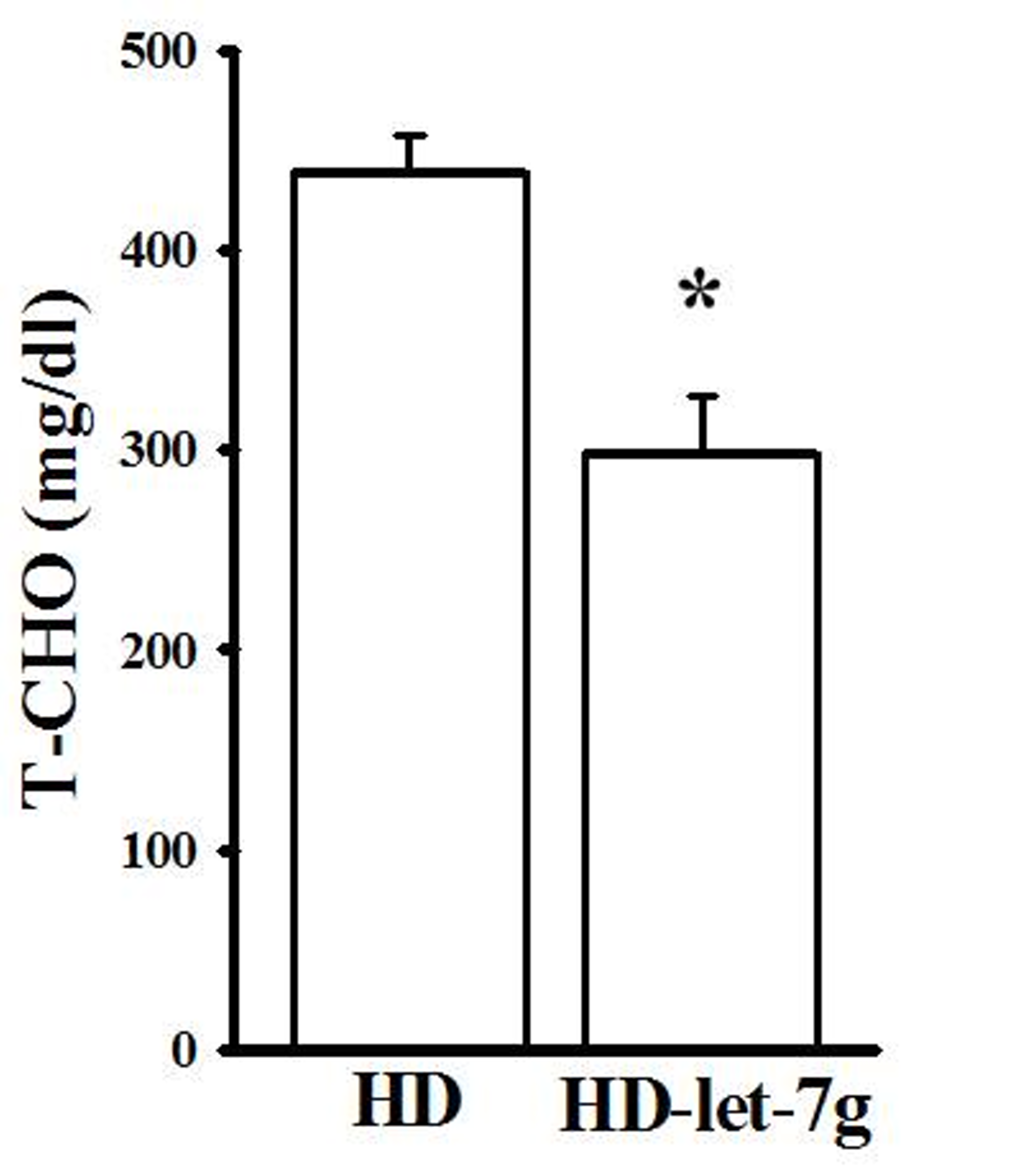

Supplement: Supplementary file 7 [file JCMM-21-3592-s006.tif]

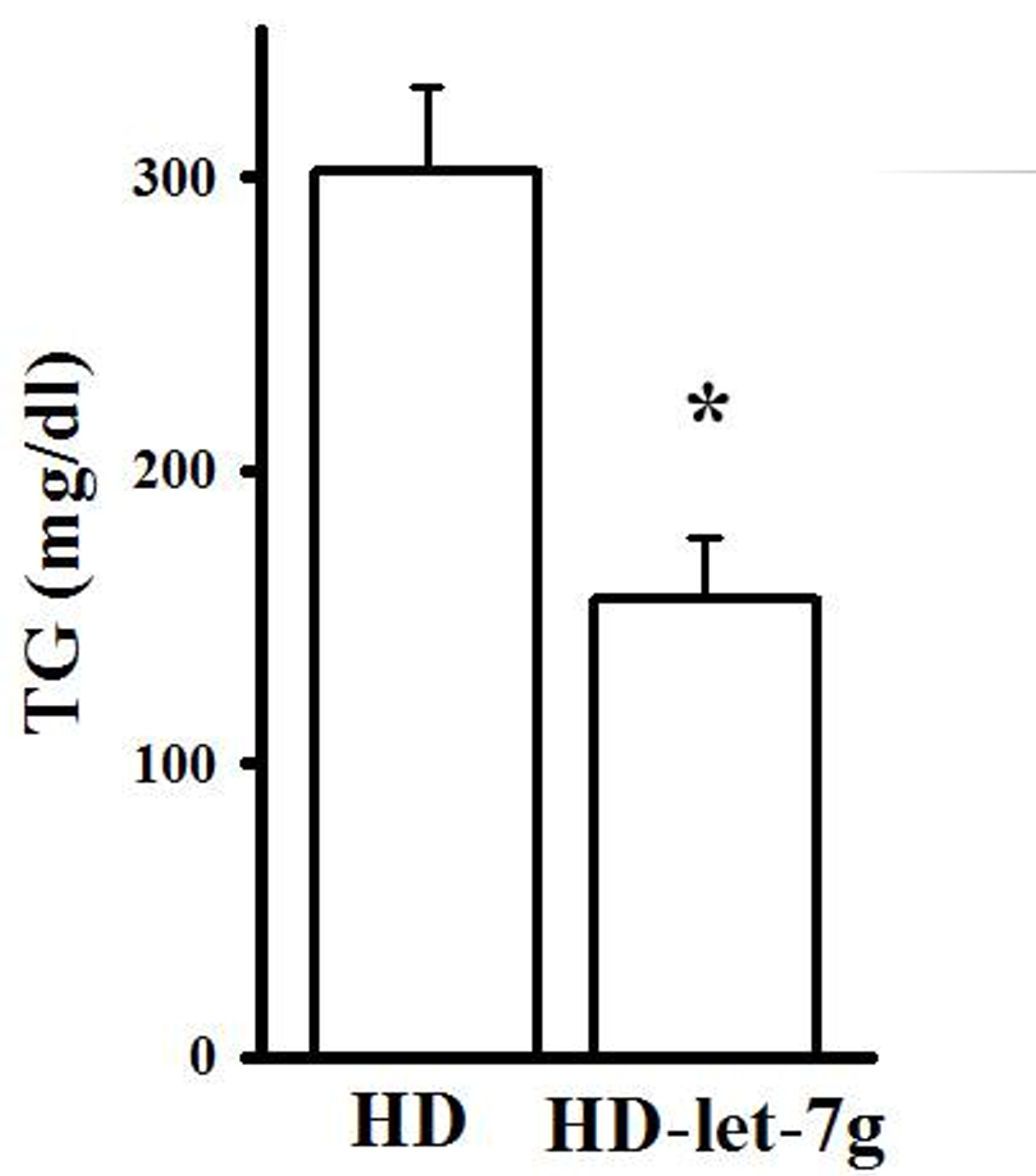

Supplement: Supplementary file 8 [file JCMM-21-3592-s007.tif]

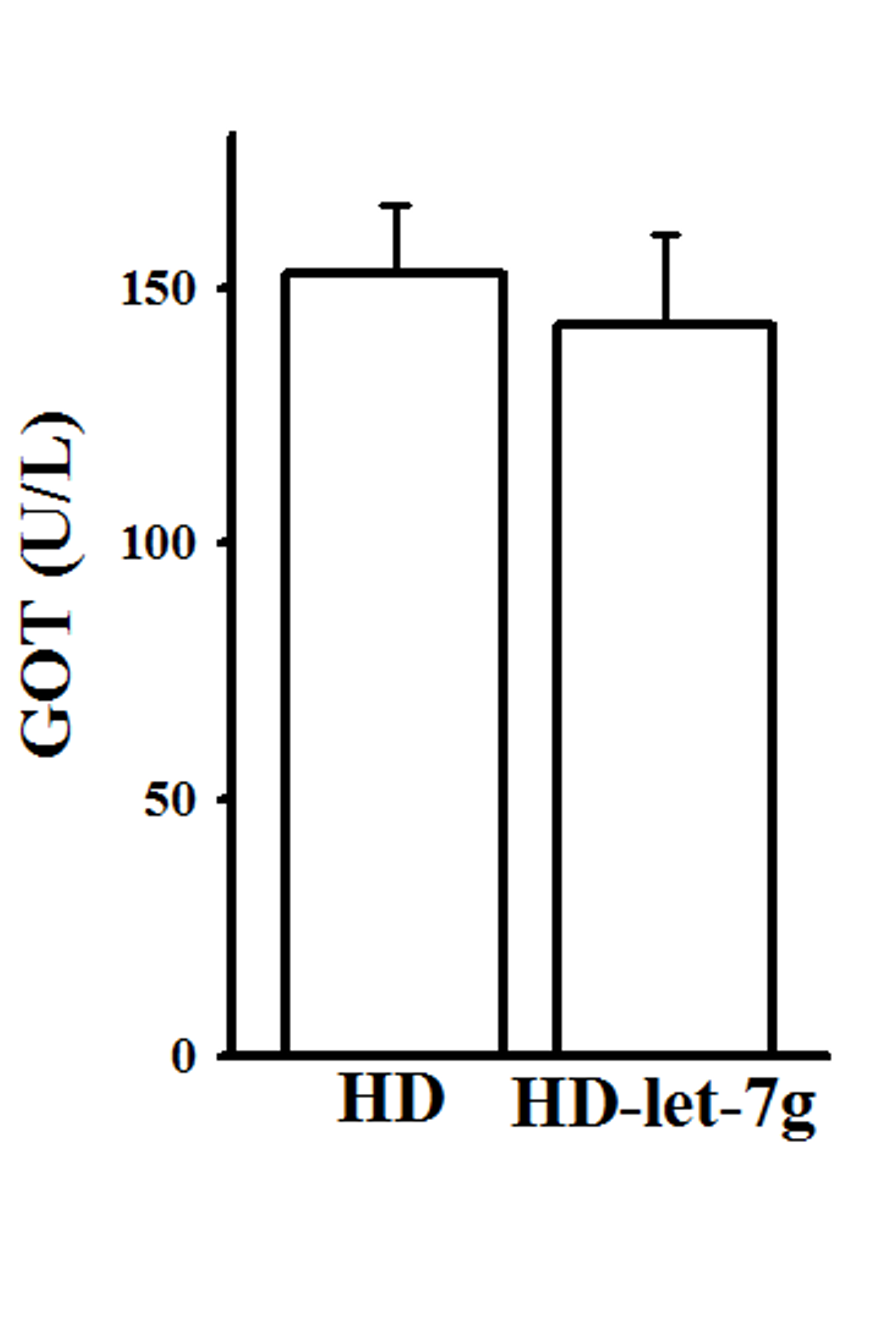

Supplement: Supplementary file 9 [file JCMM-21-3592-s008.tif]

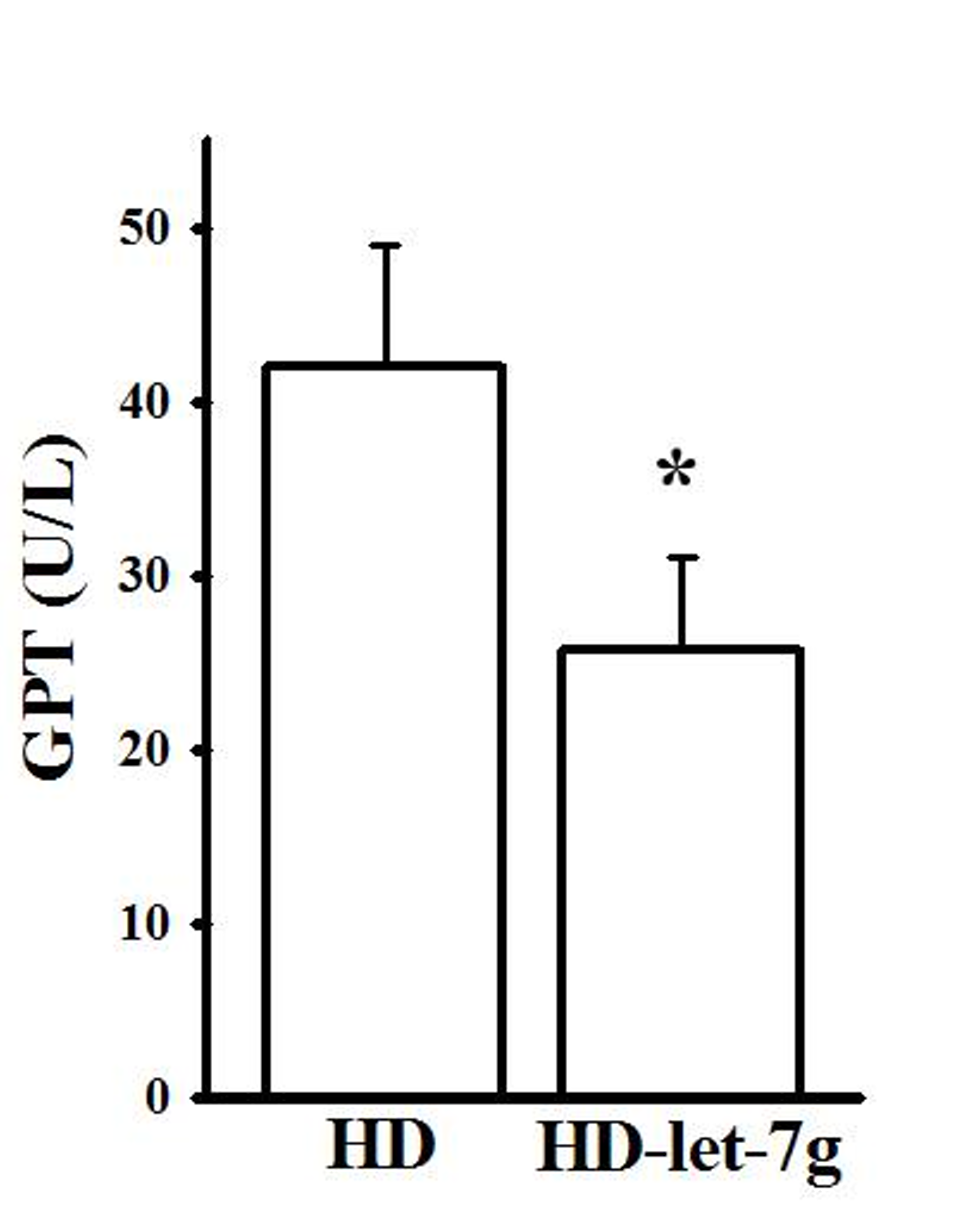

Supplement: Supplementary file 10 [file JCMM-21-3592-s009.tif]

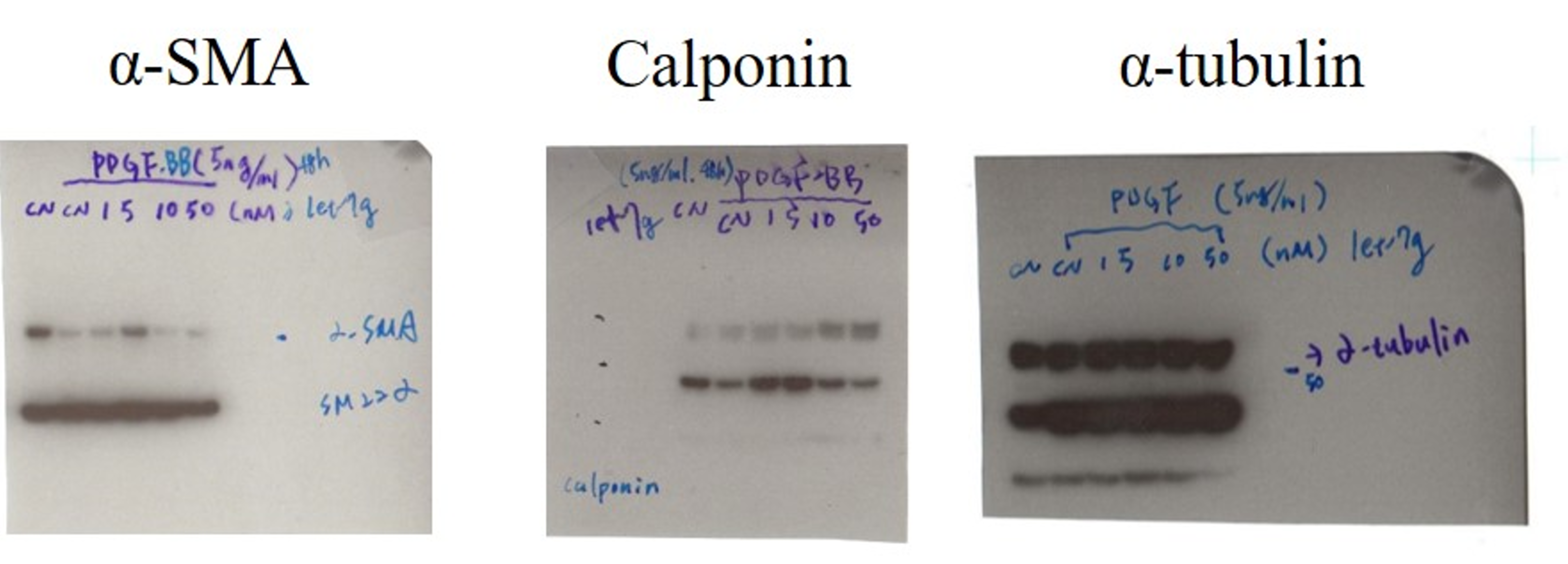

Supplement: Supplementary file 11 — Figure S2 Entire Western blotting analysis of α‐SMA and calponin. [file JCMM-21-3592-s010.tif]

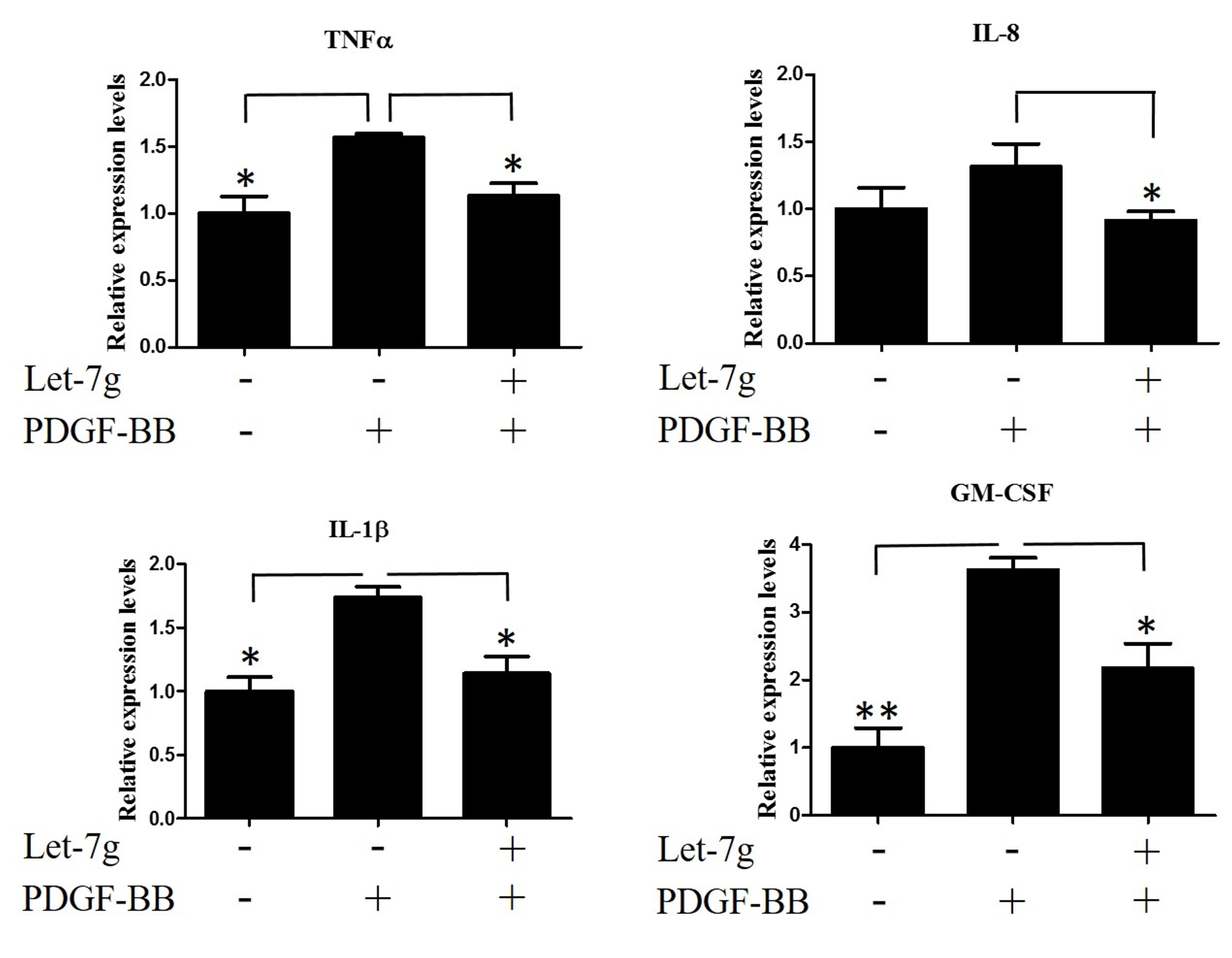

Supplement: Supplementary file 12 — Figure S3 Let‐7g inhibits inflammatory genes expression in PDGF‐BB‐treated HASMCs. [file JCMM-21-3592-s011.tif]

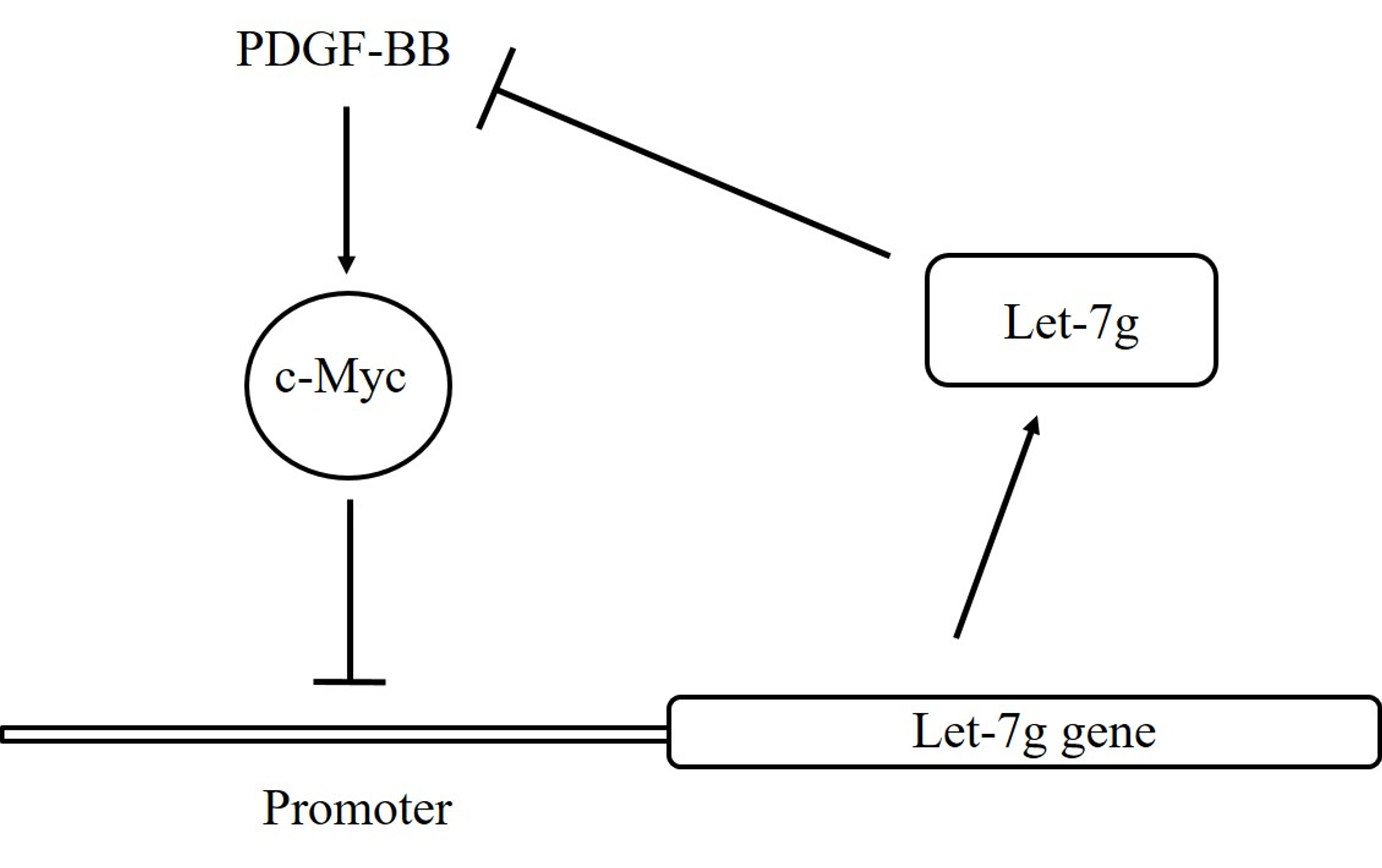

Supplement: Supplementary file 13 — Figure S4 Schematic diagram showing the possible mechanism of PDGF‐BB reduces let‐7g expression. [file JCMM-21-3592-s012.tif]
